# Photocontrolled Strain in Polycrystalline Ferroelectrics via Domain Engineering Strategy

**DOI:** 10.1021/acsami.1c03162

**Published:** 2021-04-21

**Authors:** Fernando Rubio-Marcos, Adolfo Del Campo, Jonathan Ordoñez-Pimentel, Michel Venet, Rocío Estefanía Rojas-Hernandez, David Páez-Margarit, Diego A. Ochoa, José F. Fernández, Jose E. García

**Affiliations:** † Department of Electroceramics, Instituto de Cerámica y Vidrio, CSIC, Madrid 28049, Spain; ‡ Department of Physics, Universitat Politècnica de Catalunya, BarcelonaTech, Barcelona 08034, Spain; § Department of Physics, Universidade Federal de Sao Carlos, Sao Carlos 13565-905, Brazil; ∥ Department of Mechanical and Industrial Engineering, Tallinn University of Technology, Tallinn 19180, Estonia

**Keywords:** photoferroelectrics, barium titanate, ferroelectric domains, photoinduced strain, light-induced phenomena

## Abstract

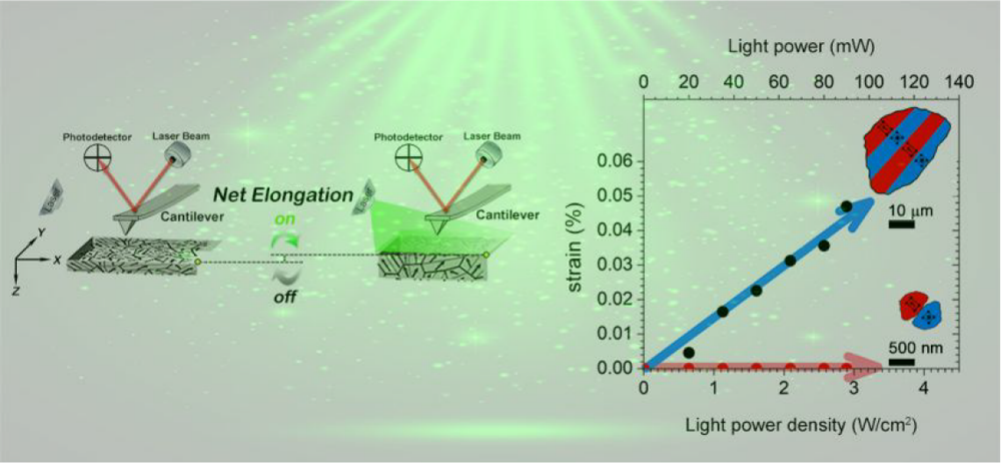

The use of photonic concepts to achieve nanoactuation based on light triggering requires complex architectures to obtain the desired effect. In this context, the recent discovery of reversible optical control of the domain configuration in ferroelectrics offers a light-ferroic interplay that can be easily controlled. To date, however, the optical control of ferroelectric domains has been explored in single crystals, although polycrystals are technologically more desirable because they can be manufactured in a scalable and reproducible fashion. Here we report experimental evidence for a large photostrain response in polycrystalline BaTiO_3_ that is comparable to their electrostrain values. Domains engineering is performed through grain size control, thereby evidencing that charged domain walls appear to be the functional interfaces for the light-driven domain switching. The findings shed light on the design of high-performance photoactuators based on ferroelectric ceramics, providing a feasible alternative to conventional voltage-driven nanoactuators.

## 1 Introduction

Control of the crystal lattice distortion in a material (that is, its strain) by light may offer the opportunity to develop new applications that are as yet unexplored.^1−10^ The appearance of new stimuli able to tune material response in a more-efficient and less-invasive manner opens up unexplored possibilities to design new device concepts. In this perspective, the reversible optical control of the macroscopic polarization in ferroelectrics has provided an excellent platform for strain control, offering notable advantages such as reduced Joule heating losses, noninvasiveness, high spatiotemporal resolution, and easy external control of the strain without physical contact.^7−9^ The electrostrain, which aims to produce a strain by the application of an external electric stimulus, is a critical aspect of materials for their use as actuators.^11−13^ Because the fundamental electrostrain mechanism is governed by domain switching in ferroelectrics,^12^ many efforts have been made to attain efficient control over it, but typically through electric fields. This concept may be radically changed by the control of the domain switching by light.^8^

The reversible light-driven domain-switching phenomenon in ferroelectric crystals appears to be related to the appearance of long-range strain fields as a result of the symmetry breaking in the domain structure provoked by the stabilization of the charged domain walls (CDWs).^14^ This ability of ferroelectrics to be photocontrolled has attracted academic as well as technological interest. To date, however, this emergent phenomenon is fundamentally associated with ideal archetypes such as single crystals, which have high-cost manufacturing on a large scale, which is a limiting factor in the rapid development of emerging light-controlled devices. In this respect, replacing ferroelectric crystals with polycrystalline materials seems to be mandatory, but conditioned so that polycrystals can develop an exploitable photoperformance. In this work, how the photostrain emerges in polycrystalline ferroelectrics is experimentally evidenced as the ferroelectric domain configuration is manipulated. In particular, it is evidenced that the photoresponse is induced by the CDWs appearance, which enables the development of a large photostrain in polycrystalline barium titanate (BTO) comparable to the conventional electrostrain values.

## 2 Experimental Section

### 2.1 Sample Preparation

Polycrystalline samples were obtained from high-purity commercial BaTiO_3_ powder (Sigma-Aldrich). The homogeneous particle size of 0.35 μm was obtained after high-energy ball-milling. The powder was heat-treated to remove moisture and then was uniaxially (350 MPa) and isostatically (250 MPa) pressed to obtain the pellets. Two sintering schemes were designed to obtain high-density (>98%) fine- and coarse-grained samples. Samples with a small grain size of 0.42 μm were obtained by sintering at 1220 °C for 8 h, while a large grain size of 42 μm was obtained by sintering at 1350 °C for 2 h. More details about the microstructure of the sintered samples are given in Supporting Information S1. The surfaces of the samples were carefully polished in two steps to avoid topographic artifacts due to nanoroughness on the sintered samples. The first step is based on a “hard polished” process using silicon carbide abrasive papers (MetaServ 250 Grinder-Polisher, Buehler, an ITW Company) and ethanol as the coolant. This was carried out on each sample to obtain parallel surfaces. In the last step, both sides of the samples were softly polished with diamond paste to obtain mirror-finish surfaces using a VibroMet 2 Vibratory Polisher (Buehler, an ITW Company), inhibiting the microroughness of the sample surface finish. The polished specimens were cut using a cutting machine (IsoMet Low-Speed Precision Cutter, Buehler), obtaining final dimensions for each sample of 5 mm × 5 mm × 0.5 mm. To remove the internal stress generated by the polishing and cutting processes, the samples were heat-treated at a temperature above the Curie temperature and then were slowly cooled. Finally, the samples were cleaned with ethanol and acetone before their characterization. No other supplementary thermal and/or chemical treatments were used to reveal the domain structure.

### 2.2 Confocal Raman Microscopy

The ferroelectric domain configuration of each sample is determinate by a Witec alpha-300RA Confocal Raman microscope. For the fine-grained samples, domain imaging in surface and cross section were performed with a 100× objective lens and a numerical aperture of 0.95 on sample regions of 10 × 10 μm and 10 × 5 μm, respectively. The Raman images revealing the domains were built from 100 × 100 and 100 × 50 spectra (that is, 10 000 and 5 000 spectra for each Raman image, respectively) with an acquisition time of 0.1 s per spectra and a laser power of 40 mW. The collected data were analyzed by using Witec Control Plus Software. For the coarse-grained sample, the analyzed regions were 70 × 70 μm on the surface and 35 × 7.5 μm in cross section, which were generated by 140 × 140 (surface scan) and 140 × 30 (depth scan) spectra using the same conditions of time acquisition and laser power.

### 2.3 Atomic Force Microscopy

Topographic characterization is mapped by the coupled atomic force microscopy (AFM) tip of the Witec alpha-300RA Raman microscope, operating in noncontact mode with a resonant frequency of 268 kHz at room temperature in an ambient atmosphere. The NSG30 AFM tip (NT-MDT, Russia) used in this study is of gold-coated silicon and is 14–16 μm high, with an aspect ratio from 3:1 to 5:1 and a typical tip radius of 10 nm. Under these conditions, AFM images were captured by scanning 256 lines with 1024 points per line at 1 Hz for each sample.

### 2.4 Local Light-Induced Photostrain

To characterize the light-induced photostrain at a local scale, a handmade optical system was designed that was coupled to AFM (Witec alpha-300RA Raman microscope). The built system allows the illumination of the BTO samples with a diode laser (Thorlabs, Inc.) operating at a wavelength of 532 nm. The power light can be continuously controlled via a linear variable ND filter, which ranges from 0 (dark condition) to 90 mW (maximum power of the light source). Under the illumination condition, the light power is calibrated by a power meter (PM100D model, Thorlabs, Inc.) integrated with an S121C silicon photodiode detector (Thorlabs, Inc.). To determine the in situ light-induced photostrain effect, the net elongation induced by off–on–off cycles of illumination is mapped in the same regions previously studied by confocal Raman microscopy (CRM) and AFM under dark conditions. The local measurements start with the AFM mapping of one-third of the total region of the sample under dark conditions (that is when laser illumination is off). When the first one-third is completed, the iris of the optical system is opened, and the second one-third of the mapping is recorded under illumination conditions. Finally, after the second one-third is finished, the iris is closed and the last one-third of the image is recorded under dark conditions. This experimental procedure allows for obtaining in situ three regions on a single AFM image that are associated with the off–on–off sequence for each sample as well as each selected light power. Each 35 μm × 35 μm AFM image is obtained by scanning 256 horizontal lines at a frequency of 1 Hz (i.e., one image requires a time of 256 s). Thus, the light is on for ∼85 s. The obtained AFM images were treated using MATLAB (The MathWorks, Inc.), and the different profiles were selected to evidence the net elongation induced by light for each sample. To test a possible thermal drift of the samples under illumination conditions, the samples are in situ monitored by a thermal camera (FLIR Systems T440) during the experiments (see ref (29) for more details). It should be pointed out that the maximum thermal drift registered under extreme illumination conditions was <3.0 °C.

### 2.5 Macroscopic Electrical Characterization

To elucidate the light-induced changes in the macroscopic dielectric permittivity of each BTO sample, the two sides of each sample were coated with ca. 200-nm-thick indium tin oxide (ITO) as electrodes and were connected to the front and back electrodes via a mechanical contact using two gold pins. The admittance of the polycrystalline samples was tested using an impedance analyzer HP4294A in the frequency range from 1 to 100 kHz at room temperature. The experimental conditions for light-stimulated experiments are the same as those previously used in light-induced photostrain measurements at a local scale. The laser spot diameter was adjusted at ∼2 mm. More details about the experimental setup for photocapacitance measurements can be found in a previous work.^15^

## 3 Results and Discussion

### 3.1 Designing Ferroelectric Domain Structures

By considering CDWs as functional elements for the photoresponse observed in polycrystalline ferroelectrics,^7^ the configuration of the domain structure is proposed to be manipulated in two extreme cases through grain-size control. In terms of the grain-size effects, BTO-based materials are one of the most studied. According to previous studies, polycrystalline BTO with submicron grain size is composed of substantially single-domain, highly stressed grains.^16−18^ By contrast, when the grain size is larger, twinning through the formation of both 180° and 90° domain walls are formed in the tetragonal phase to minimize residual stress (that is, large grains usually develop a multidomain configuration at room temperature).^18^ Fine- and coarse-grained BTO samples were then sintered, having average grain sizes of 0.42 and 42 μm, respectively.

**Figure 1:**
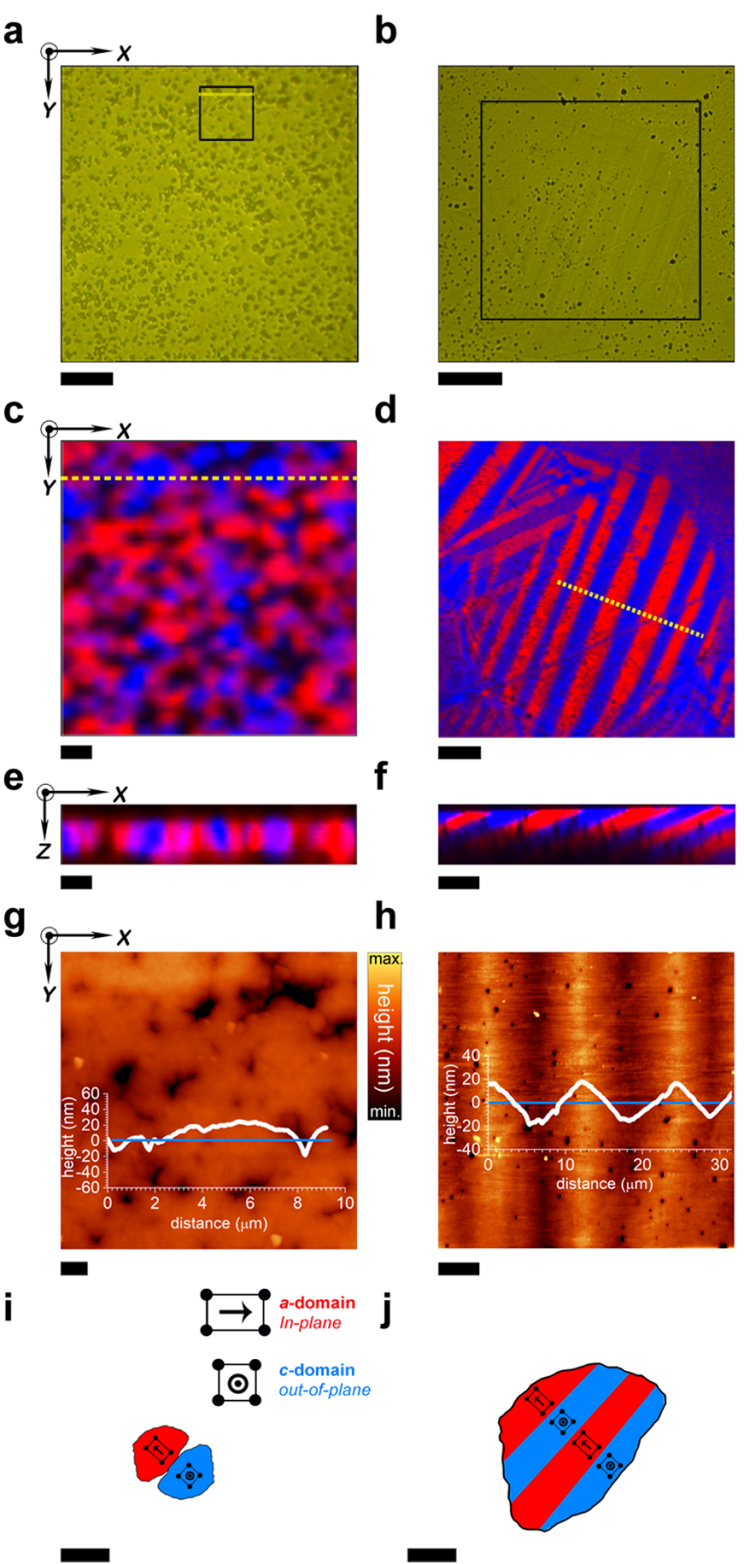
Identification of the domain structure of BTO polycrystals. (a, b) Optical images of the surface of the fine-grained (a) and coarse-grained (b) BTO samples. Scale bars, 10 and 20 μm, respectively. The regions indicated by a black square and white dashed arrow show the positions where the surface (c, d) and cross-sectional (e, f) confocal Raman images are taken for each sample. Scale bars in panels (c) and (e) correspond to 1 μm, while those in panels (d) and (f) correspond to 10 μm. The fine-grained BTO sample shows a single-domain-type configuration (c, e), while the coarse-grained sample is composed of an *a/c/a-*like domain configuration (d, f). The average Raman spectra correspond to *a-*domain, i.e., in-plane polarization (red spectrum), and the identifications of *c-*domain, i.e., out-of-plane polarization (blue spectrum), can be examined in Supporting Information S2. (g, h) AFM images of each sample. Scale bars, 1 and 5 μm, respectively. The topography along the yellow dashed lines marked in panels (c) and (d) is shown over each AFM image. (i, j) Schematic summary of the domain configuration developed for each sample. Scale bars, 500 nm and 10 μm, respectively.

The emergence of the different ferroelectric domain configurations as a result of grain-size dissimilarity is determined by the simultaneous combination of confocal Raman microscopy (CRM) and atomic force microscopy (AFM) imaging (Figure 1). CRM images show different ferroelectric domain configurations as the grain size is varied (Figure 1c–f). Note that the spatial maps of the CRM intensity reveal the presence of the two types of ferroelectric domains in the samples (red or blue in the image), with their contrast being generated by the different Raman spectra (Supporting Information S2). Consequently, red and blue spectra correspond to in-plane polarized domains (*a*-domains) and out-of-plane polarized domains (*c*-domains), respectively. As expected, the CRM spatial maps confirm that fine-grained BTO (Figure 1c and e) is composed of substantial grains with a single direction of polarization (i.e., single or 180° domains), while the coarse-grained sample (Figure 1d and f) develops *a/c/a-*like domains (usually called 90° twins) inside the grains, where the in-plane and out-of-plane polarization components alternate 90° to the neighboring stripe domains.^19^

The topographical features of the samples at the local scale, in the same regions previously studied by CRM (Figure 1g and h), are characterized by AFM. Figure 1g shows the surface topography of the fine-grained sample, which is quite flat with a small roughness in the range between 10 and 20 nm as a result of the polished process. As expected, a different morphology is revealed for the coarse-grained sample, as shown in Figure 1h. The AFM scan reveals the domain boundary topography associated with asymmetric sawtooth transitions. The ferroelectric domain walls are determined by the reversal of the inclination angle in the topographic profile. The topography features together with the Raman data unveil the domain configuration (see Supporting Information S3 where a representative scheme of the domain structure for the coarse-grained sample is built), showing the emergence of two different types of 90° domain walls (that is, the *a*/*c*- and the *c*/*a*-domain walls are not morphologically symmetric and not energetically equivalent). The polarization vectors in neighboring domains can be organized either head-to-head (H–H), resulting in strongly charged domain walls (sCDWs), or tail-to-tail (T–T), forming weakly charged domain walls (wCDWs).^20,21^ In accordance with previous observations on single crystals, the *a/c*-domain wall (Figure S3a–c) is constituted of in-plane and out-of-plane polarizations alternating 90° to the neighboring stripe with a H–H configuration of the polarization vectors (the sCDWs are located at the peak zone of the topography profile).^19^ Conversely, the *c/a*-domain wall has a T–T configuration of the polarization vectors (located on valley regions of the topography profile). Hence, two extreme ferroelectric domain structures have been designed through grain size control. Results from the classical electric-field-induced polarization (P-E) and strain (S-E) measurements are in accordance with the previous results attained by CRM and AFM related to the domain configurations of fine- and coarse-grained BTO, as shown in Supporting Information S4.

### 3.2 Light-Induced Strain at a Local Scale

**Figure 2:**
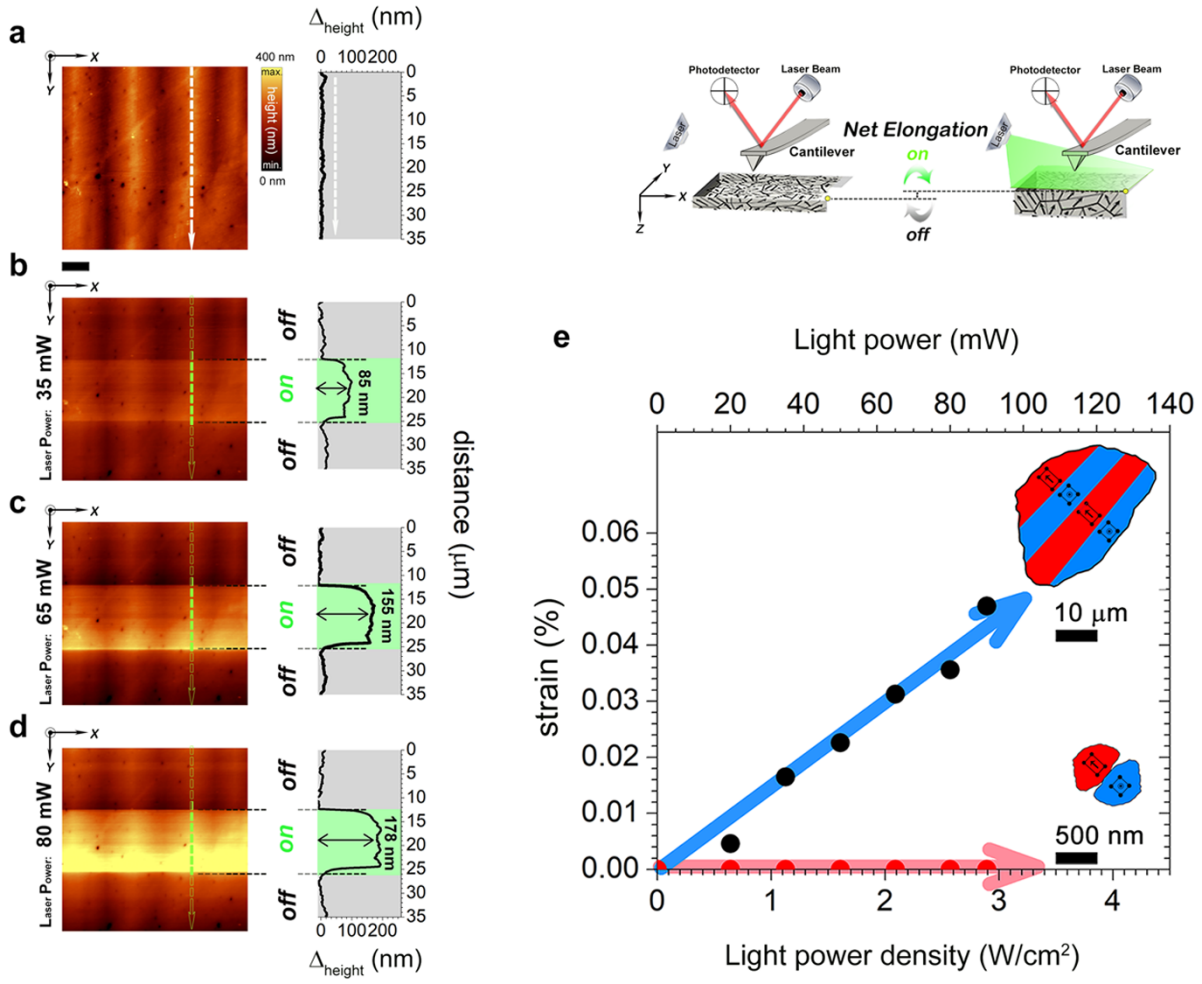
Light-power density dependence of strain in BTO polycrystals. (a–d) Set of AFM images revealing the reversible net elongation under different light powers for the coarse-grained BTO sample. Scale bar, 5 μm. The topography profile is plotted inside each AFM image along the white arrow (dark condition) and the green dashed arrows (illumination condition). (b, c) An off–on–off light cycle is performed in all cases. The value of the light power is indicated on the left of each AFM image. Additionally, a simplified experimental scheme is shown in the right part of image (a). (e) Evolution of the strain as a function of both light-power density and light power for each sample. The red and blue curves represent the fine- and coarse-grained BTO samples, respectively. The strain is calculated according to the thickness of the samples (500 μm for both samples). The error bars are not visible because the symbol size is greater than the error obtained from the standard deviation, which was calculated by measuring the photoinduced strain in >10 grains. The light-power density was calculated according to the light spot diameter, which is 2 mm. The domain configuration schemes for each sample have been included in the plot for clarity.

Given that electric-field-induced strain manifests a clear dependence with domain configuration and that reversible photocontrol of the domain switching in BTO single crystals seems to be intimately related to the stabilization of the CDWs,^8,9^ the next step should be to explore if the polycrystalline materials are photostrain tunable, and more importantly if they can develop a similar level to the single crystal’s photoperformance. An AFM coupled to a handmade optical system is used to characterize the light-induced strain at a local scale. The BTO samples are illuminated by using a green laser diode with a wavelength of 532 nm (i.e., a photon energy of 2.3 eV) and variable light power. The topography profiles acquired in illumination conditions (Figure 2a–c) evidence a local net photoelongation that increases almost linearly with light power, thereby revealing the photosensitive nature of the coarse-grained BTO. However, no photostrain response is detected for the fine-grained sample under the same illumination conditions (red arrow in Figure 2d), strengthening the pivotal idea that this emergent phenomenon is dominated by the appearance of CDWs.

Focusing on the coarse-grained BTO, the photostimulation induces a reversible strain with a net elongation of ca. 235 nm under a light-power density ca. 3 W cm^–2^ (light power of 90 mW), which implies a remarkable photoinduced strain value of ca. 0.05%. Specifically, the photostrain value obtained is comparable with the conventional electrostrain value obtained under an applied electric field of 1 kV mm^–1^, taking the optical-control relevant advantages over traditional electric-field stimulus because it means a contact-less approach (Supporting Information S5 shows a direct comparison between the photo- and electrostrain values). Furthermore, it is important to point out that the achieved photostrain in coarse-grained BTO is comparable to the previously reported photostrain in BTO crystals,^9^ allowing for development of new photocontrolled devices at a lower cost.

### 3.3 Light-Controlled Macroscopic Response

**Figure 3:**
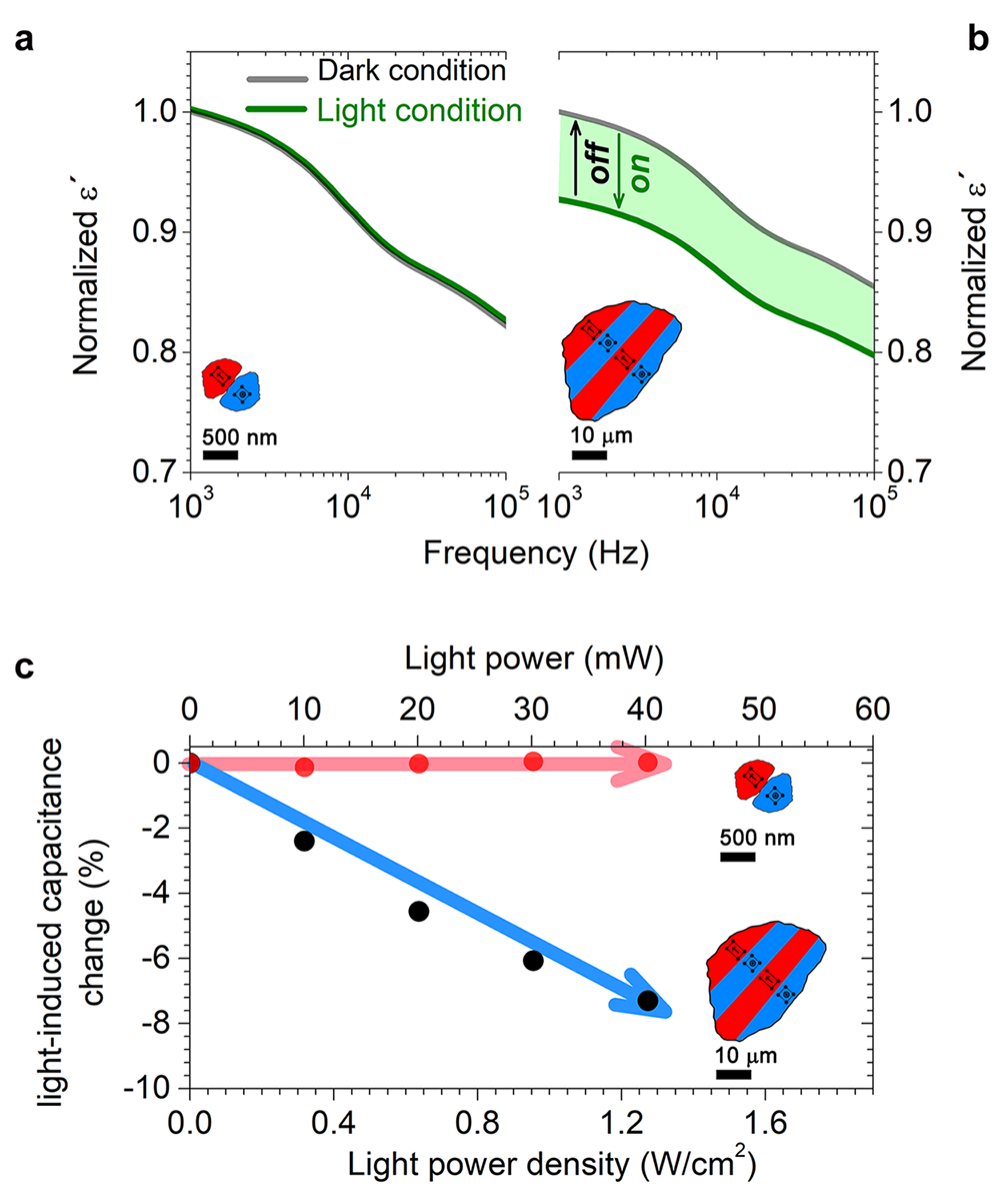
Experimental proof of the long-range photoresponse in BTO polycrystals by monitoring the macroscopic dielectric response. (a, b) Real permittivity, ε′, of fine-grained (a) and coarse-grained (b) BTO samples under dark and illumination conditions. In both cases, the ε′ values are normalized by taking into account the value in the dark condition at 1 kHz. Note that no variation of ε′ is detected for the fine-grained sample. By contrast, a remarkable change in the real permittivity is shown in a coarse-grained sample as a result of a light-induced change in the domain configuration. The percentage of light-induced capacitance change is represented by the green region in panel (b). The scheme shown inside each figure represents the two different domain configurations. (c) Comparison of light-induced capacitance change between the two extreme cases under study as a function of both light-power density and light power, where the red and blue curves depict the fine- and coarse-grained BTO samples, respectively. The light-power density was calculated according to the light-spot diameter, which is 2 mm.

The functionality of the material is subject to its ability to be able to produce a macroscopic response that can be easily monitored. Therefore, macroscopic real permittivity, ε′, is measured in dark and illumination conditions to confirm that a macroscopic light-induced functional response there exists. Figure 3 illustrates the change in ε′ upon multiple off–on–off cycles of illumination for both samples. No significant difference is seen in the ε′ values when the fine-grained sample is photostimulated (Figure 3a), which is consistent with the previous results. In contrast, ε′ undergoes an evident change of ca. 8% for coarse-grained samples under the same experimental conditions (Figure 3b). Note that ε′ returns to its initial value whenever the illumination is off, thereby proving that the light-driven domain rearrangement is a reversible effect. Figure 3c summarizes the light-power dependence of the light-induced capacitance change for the two extreme cases under study. Again, it is evident that domain configuration is a key factor in the emergent photocontrol of functional properties in ferroelectrics. The light power dependence reveals a clear linear dependence as occurs on local light-induced photostrain (Figure 2d), which is relevant because there is not a light-power threshold.

### 3.4 Discussion

On the basis of our observations and some recent results of other authors,^7,22^ some intriguing features can be discussed. First, note that the photovoltaic effect can be discarded as the primary physical origin of this phenomenon because it requires illumination conditions with photon energy higher than the BTO bandgap to generate the desired photoexcited carrier separation. However, recent studies suggest that the bandgap may decrease up to 20% at the domain wall.^23^ Therefore, we have performed a new measurement of the photostrain under one additional wavelength of the light source (Supporting Information S6). The selected light wavelength was 658 nm, even further from the sample bandgap, in order to definitively avoid the photovoltaic effect. The results shows that no significant differences are exhibited between the net elongation induced by a red (658 nm) and a green (532 nm) light under the same illumination conditions (50 mW and 2 mm spot diameter), thereby endorsing that the photoresponse is wavelength-independent in the visible range in a similar manner to how it was demonstrated for BTO crystals.^15^ Second, the thermal expansion can also be discarded as the fundamental origin of the observed photoresponse because the sample’s temperature increases up to a maximum of 3 °C for the maximum light power used. Therefore, on the basis of the thermal expansion coefficient of the BTO, the thermal-induced strain for such thermal drift turns out to be 1 order of magnitude smaller than the light-induced strain. Third, the CDWs emerge on the coarse-grained sample, but they do not appear in the fine-grained sample (grain size <0.5 μm). When grain size is low enough, the grain boundaries restrict the formation of non-180° domains. Fourth, the CDWs stabilization originates the alteration of the energy bands in the coarse-grained BTO, creating an anisotropy of the potential at domain boundaries with a saw-tooth morphology.^23−25^ The accumulated charge at CDWs appears to compensate for the bound polarization charges from the domains to make the net pressure over the wall zero.^26^ This charge exhibits metallic conductivity and consequently can be regarded as “locally free charges”, similar to a 2D electron gas at interfaces.^27^ Under these boundary conditions, an alternating external force (that is, light as a stimuli source) produces domain-wall motion through the so-called ratchet effect,^28,29^ thereby resulting in a net elongation of the system (that is, photostrain response). This phenomenon should be observable in any ferroelectric material that shows charged domain walls, as was recently evidenced.^7^ Finally, additional results show that the contribution of the Ba–O bond to the photoresponse depends on the domain type. Under illumination conditions, the Ba–O bond contributes more to the polarization than the Ti–O bond in the *a*-domain, while a lower Ba–O bond contribution is detected in *c*-domains, thereby generating a polarization imbalance in the system (Supporting Information S7). This fact would reinforce symmetry breaking, which is related to the appearance of long-range strain fields,^14^ and consequently provide evidence of a relationship between lattice-charge coupling and its possible photocontrol at both micro- and macroscopic levels.

## 4 Conclusions

In summary, light illumination on BTO ceramics is found to act as a virtual electrical field, and the charge injection/modulation is governed by the charged domain walls stabilization, which can be manipulated via a domain-engineering strategy. The results indicate that an adequate domain-engineering strategy plays a fundamental role in the photofunctional response of polycrystalline ferroelectrics, with the CDWs stabilization being mandatory. Under these boundary conditions, the attained photostrain in ferroelectric polycrystals proves to be at the same level of the more complex archetype systems, allowing the development of new photocontrolled devices at a lower cost. It is worth mentioning that the phenomenon is evidenced not only at the microscopic scale but also at the macroscopic level. From a broader perspective, this work contributes to extending the fundamental knowledge based on light-ferroic coupling, opening new opportunities to speed up the development of a new generation of contact-less photoelectronic devices.

## Supporting Information Available

Basic identification of the morphology and crystal structure of BTO samples, determination of the polarization configuration from CRM, schematic representation of the domain configuration of the coarse-grained BTO by the simultaneous combination of CRM and AFM imaging, ferroelectric and strain responses of the studied BTO samples, comparison between electrostrain and photostrain responses in coarse-grained BTO, and influence of the lattice-charge coupling in the photoresponse phenomena of polycrystalline ferroelectrics (PDF)
