# Supplementary material for: Photocontrolled Strain in Polycrystalline Ferroelectrics via Domain Engineering Strategy
Source: ACS Appl Mater Interfaces. 2021 Apr 21;13(17):20858–64. doi: 10.1021/acsami.1c03162 (PMC8480775; doi:10.1021/acsami.1c03162)
Supplement: Supplementary file 1 — am1c03162_si_001.pdf [file am1c03162_si_001.pdf]

Supporting Information

Photo-Controlled Strain in Polycrystalline Ferroelectrics via Domain Engineering Strategy

Fernando Rubio-Marcos,[a,] Adolfo Del Campo,[a] Jonathan Ordoñez-Pimentel,[b,c] Michel Venet,[c] Rocío Estefanía Rojas-Hernandez,[d] David Páez-Margarit,[b] Diego A. Ochoa,[b] José F. Fernández,[a] and José Eduardo García[b,*]*

[a] *Department of Electroceramics, Instituto de Cerámica y Vidrio - CSIC, 28049 Madrid, Spain.*

[b] *Department of Physics, Universitat Politècnica de Catalunya, 08034 Barcelona, Spain.*

[c] *Department of Physics, Universidade Federal de Sao Carlos, 13565-905 Sao Carlos, Brazil.*

[d] *Department of Mechanical and Industrial Engineering, Tallinn University of Technology, 19180 Tallinn, Estonia.*

Corresponding Authors

*E-mail: frmarcos@icv.csic.es (F.R-M.).

*E-mail: jose.eduardo.garcia@upc.edu (J.E.G.).

S1. Basic identification of the morphology and crystal structure of BTO samples.

To validate the efficiency of the synthesis strategies on the design of different ferroelectric domain configuration via grain size control, the microstructures of both samples are characterized by using a Field Emission Scanning Electron Microscope, FE-SEM (Hitachi S-4700). It is found that the sintering strategy has a notorious impact on the grain (**Figures 1Sa-b**). The average grain size (localized inside each SEM image) is found to increase from 0.4 μm to 40 μm, which suppose the difference in the grain size of two orders of magnitude. Thus, the samples provide two extreme scenarios, in which the ferroelectric domain configurations are expected to be radically different.

The crystal structures were examined by using X-ray diffraction (XRD, X'Pert PRO Theta/2theta, PANalytical, The Netherlands) at room temperature (**Figures S1c-d**). XRD patterns show the stabilization of a tetragonal *P4mm* symmetry (see inserts of **Figures S1c-d**) for both samples. It may be observed, the diffraction peaks are localized in the same two-theta positions for both samples, thereby revealing that grain size has a minimal impact on the crystallographic phase.

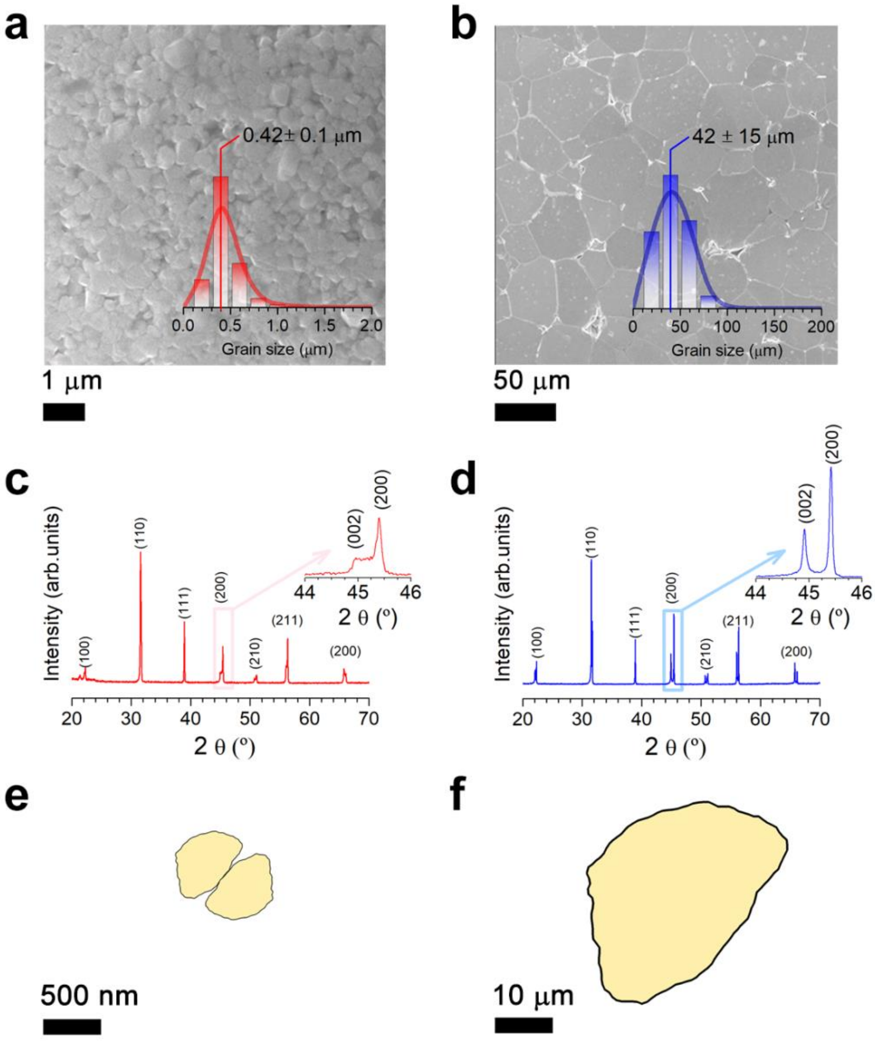

Figure S1 | Basic identification of the morphology and crystal structure of BTO samples. a-b, FE-SEM images showing the microstructure of fine-grained (panel **a**) and coarse-grained (panel **b**) BTO samples. Inside each SEM image, grain size distributions are plotted. **c-d**, X-ray diffraction patterns for fine-grained (panel **c**) and coarse-grained (panel **d**) BTO samples. The insets of the panels **c** and **d** show a detail of the XRD diffraction patterns in the 2Θ range 44° to 46°, corresponding to (002) and (200) peaks of the tetragonal symmetry. Significant diffuse scattering is displayed for fine-grained BTO, which is a common feature of polycrystals showing small grain size. **e-f**, Schematic summary of the grain size developed for each sample.

S2. Determination of the polarization configuration from CRM.

Figure S2 shows the main Raman spectra extracted from confocal Raman imaging. These average spectra are associated with different types of domains in BTO.[1,2] To determinate the polarization configuration from CRM imaging, we have been taken into account that the in-plane polarized *a*-domains are easily recognizable since they are distinguished by the vanishing of two Raman modes (that is, by the vanishing of Raman modes marked as 4 and 6 in **Figure S2**). Therefore, red and blue spectra can be assigned to the in-plane *a*-domains and to the out-of-plane *c*-domains. Additionally, the assignments of the observed Raman modes, both symmetry, and nature (first and second-order) are summarized in **Table S1**, and their average Raman spectra can be observed in **Fig. S2**.

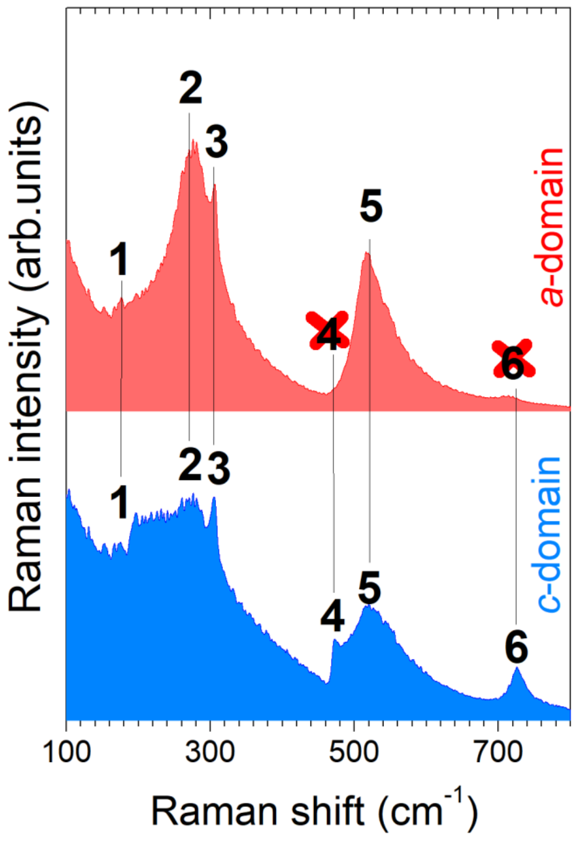

Figure S2 | Determination of polarization configuration in BTO from the Raman spectra. The average Raman spectra correspond to *a*-domain, i.e. in-plane polarization (red spectrum), and *c*-domain, i.e. out-of-plane polarization (blue spectrum) of BTO. Average spectra were extracted from Raman imaging and were processed with Witec Control Plus Software.

Table S1 | Raman modes and their mode symmetry assignments in tetragonal $BaTiO_3$ single crystal. The table summarizes both symmetry and nature (first and second-order) of the Raman modes of the $BaTiO_3$ phase. According to the nuclear site group analysis, Raman active phonons of the tetragonal *P4mm* (C_{4v}^1) crystal symmetry are represented by $3A_1 + B_1 + 4E$. Long-range electrostatic forces induce the splitting of transverse and longitudinal phonons, which results in split Raman active phonons represented by $3[A_1 (TO) + A_1 (LO)] + B_1 + 4[E (TO) + E (LO)]$.

Raman Shift (cm^{-1})	Symmetry	Abbreviated number	Reference
36	E (TO)	1	3-7
170	A_1 (TO)		4-9
180	E (TO_2), E (LO)		3-7
185	A_1 (LO)		4-9
210-270	A_1 (TO_2)	2	4-9
305	E (TO_3 + LO_2)	3	3-7
305	B_1		1-5
463	E (LO_3)	4	3-7
475	A_1 (LO_2)		4-9
486	E (TO_4)		1-5
518	E (TO_5)	5	3-7
520	A_1 (TO_3)		4-9
715	E (LO_4)	6	3-7
720	A_1 (LO_3)		4-9

S3. Schematic representation of the domain configuration of the coarse-grained BTO by the simultaneous combination of CRM and AFM imaging.

To better understand the formation of the not energetically equivalent 90° domain walls (DWs) in the coarse-grained BTO, a representation scheme of the domain structure is built in **Figure S3** from the correlative CRM (**Figure 3Sa**) and AFM (**Figure 3Sb)** imaging. The *a/c*-DW is constituted of in-plane and out-of-plane polarization components alternate 90° the neighboring stripe with a head-to-head (H-H) configuration of the polarization vectors as illustrated in **Figure S3c.** The H-H strongly charged domain wall (sCDW) is located at peak zone of the topography profile (**Figure 3Sb**), which arises due to strong mechanical stress driving the alleviation of the elastic energy into the domain wall. By contrast, the *c/a*-DW has a tail-to-tail (T-T) configuration of the polarization vectors that is situated on valley regions of the topography profile (**Figure S3b**), giving rise to a less stress degree. A more detailed explanation about the underlying mechanism contributing to the organization of polarization vectors can be found in previous work.[2]

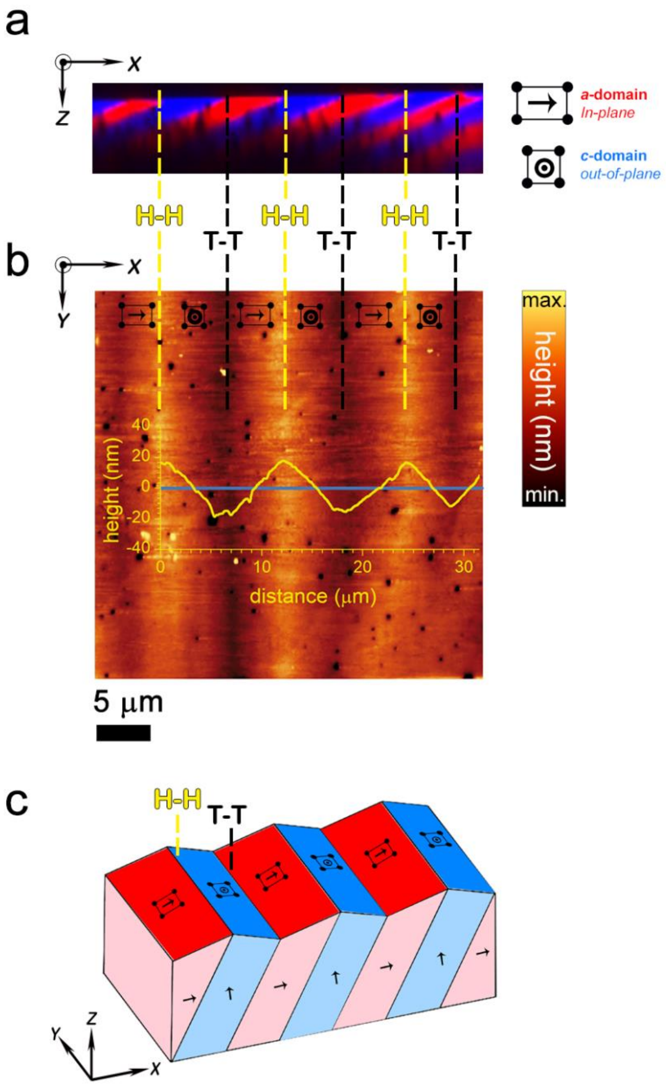

Figure S3 | Determination of the domain configuration of the coarse-grained BTO. a, Depth Raman image of the coarse-grained BTO sample. **b**, the topographic AFM image of the coarse-grained BTO sample in the same region previously studied by CRM (panel **a**). **c**, Schematic representation of the domain configuration of the coarse-grained BTO sample built from the simultaneous combination of CRM and AFM imaging. Note that polarization vectors in neighboring domains can either be organized head-to-head (marked in yellow and represented as H-H), resulting in strongly charged domain walls (sCDWs) or tail-to-tail (signaled in black color and indicated as T-T), creating weakly charged domain walls (wCDWs).

S4. Ferroelectric and strain responses of the studied BTO samples.

Macroscopic polarization (Figure S4a) and strain (Figure S4b) responses induced by a bipolar electric field were simultaneously measured for both fine- and coarse-grained BTO samples. A typical ferroelectric *P-E* loop is shown for the coarse-grained BTO, while no ferroelectric response is displayed in the fine-grained sample under the same maximum electric field. This fact is directly related to the different domain configurations. The 90° domains easily switch under a moderate electric field while single domains (also 180° domains) require high electric fields (even higher than the electrical breakdown of the material) to effectively switch. The observed difference in the electric field-induced strain for each BTO sample (Figure S4b) is also a direct consequence of the different domain configurations. The most prominent feature is that no noticeable electric field-induced strain is detected in the fine-grained sample, which is in agreement with the results obtained by CRM and AFM. When ferroelastic (that is, non-180°) domains are absent, no electric field-induced strain is expected, as shown in fine-grained BTO sample. On the contrary, the electric field induces a strain degree of approximately 0.08 % at 1.5 kV mm^{-1} for the coarse-grained BTO sample, which shows to be a consequence of the 90° domain switching.

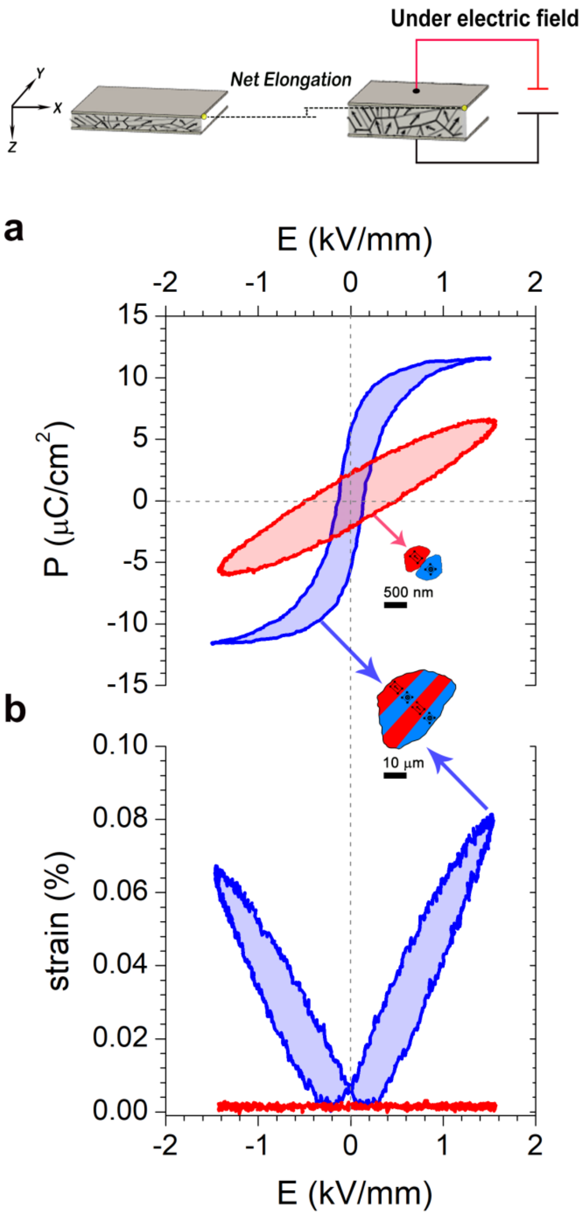

Figure S4 | Polarization- and strain-induced electric field measurement. a, Electric field-induced polarization of fine-grained (red curve) and coarse-grained (blue curve) BTO samples. A typical *P-E* hysteresis loop is displayed by coarse-grained BTO sample while a non-ferroelectric, RC behavior is shown by the fine-grained BTO sample. **b,** Electric field-induced strain of fine- and coarse-grained BTO samples. A well-known *S-E* loop is shown for coarse-grained BTO sample while no strain response is observed for fine-grained BTO one. The loops were measured at 1 Hz and under a maximum electric field of 1.5 kV/mm. The domain configuration schemes for each sample have been included inside the images for clarity.

S5. Comparison between electro-strain and photo-strain responses in coarse-grained BTO.

To demonstrate the feasibility of the photo-strain response, a comparison between it and the conventional electro-strain response is shown in **Figure S5**. As may be observed, the photo-strain values are comparable to the conventional electro-strain values, thereby supporting that the photo-strain phenomenon in ferroelectric polycrystals may be used for the development of the new generation of contactless nano-optomechanical devices.

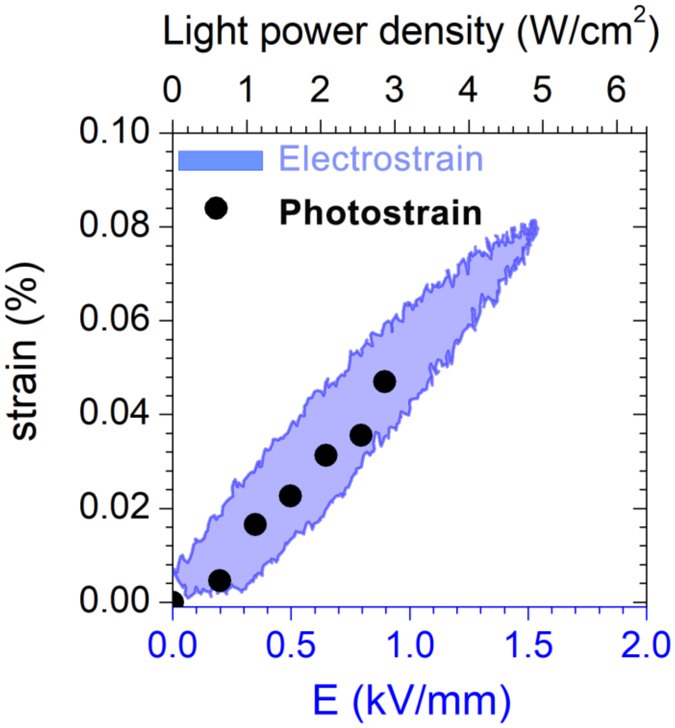

Figure S5 | Electro-strain and photo-strain responses of coarse-grained BTO. Comparative representation of the electric-field induced strain (electrostrain) and the light-induced strain (photostrain) of coarse-grained BTO sample. A typical unipolar electric-field induced strain is revealed for a maximum applied the electric field of 1.5 kV/mm. The light power density dependence of the light-induced strain shows a linear behavior. The scale of the light power density was adjusted in order to easily compare the light power and electric field needed to produce the same strain on the sample.

S6. Wavelength-independent photo-strain.

A reference measurement of the photo-strain under one additional wavelength of the light source in coarse-grained BTO was performed. Note that the light wavelength selected for this additional experiment was 658 nm, even further from the sample bandgap, to avoid the photovoltaic effect (we have ruled out the use of the wavelength lower than 450 nm because their possible proximity to the absorption spectra of the studied samples). **Figure S3** shows that no significant differences are exhibited between the net elongation induced by a red (658 nm) and a green (532 nm) light under the same illumination condition (50 mW and 2 mm spot diameter), thereby endorsing that the photo-response is wavelength-independent in the visible range.

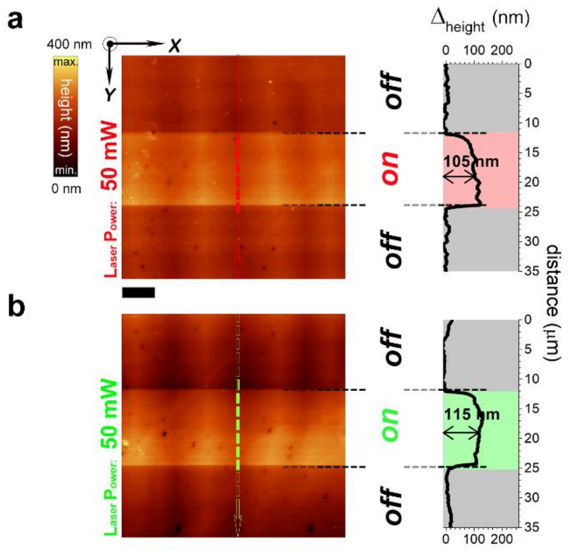

Figure S6| Wavelength dependence on the light-activated strain. The sequence of AFM images displaying the reversible strain under different visible light sources: (**a**), 658 nm and (**b**) 532 nm. In both cases the incident light power and the light spot diameter were 50 mW and 2 mm, respectively. Scale bar, 25 μm.

S7. Influence of the lattice-charge coupling in the photo- response phenomena of polycrystalline ferroelectrics.

The light-induced domain wall motion is clearly related to the lattice-charge coupling, such as the bond charge associated to the contribution of the Ba-O bond and/or Ti-O bond of the perovskite structure to total polarization of each domain.[10] In order to show the difference contributions of the Ba-O bond and/or Ti-O bond to total polarization of each domain, a comparison between the Raman spectra corresponding to *a*-domains (i.e., in-plane polarization, red region of the **Fig. S7a-b**), and *c*-domains, (i.e. out-of-plane polarization, blue region of the **Fig. S7a-b**) of coarse-grained BTO under dark and illumination conditions is introduced (**Figure S7**). The starting point is based on the fact that Raman intensity is proportional to the polarizability[11] of the both Ba-O (purple region of the panels **c** and **d** of the **Fig. S7**) and Ti-O bonds (yellow region of the panels **c** and **d** of the **Fig. S7**). Consequently, the polarizability changes induced by light can be revealed in the Raman spectra (**Fig. S7 c-d**). Under illumination conditions (that is, under a light power of 40 mW), the Ba-O bond contributes more to the polarization than the Ti-O bond in *a*-domains (**Fig. S7 c**), while a lower Ba-O bond contribution is detected in *c*-domains (**Fig. S7 d**), thereby generating a polarization imbalance in the system.

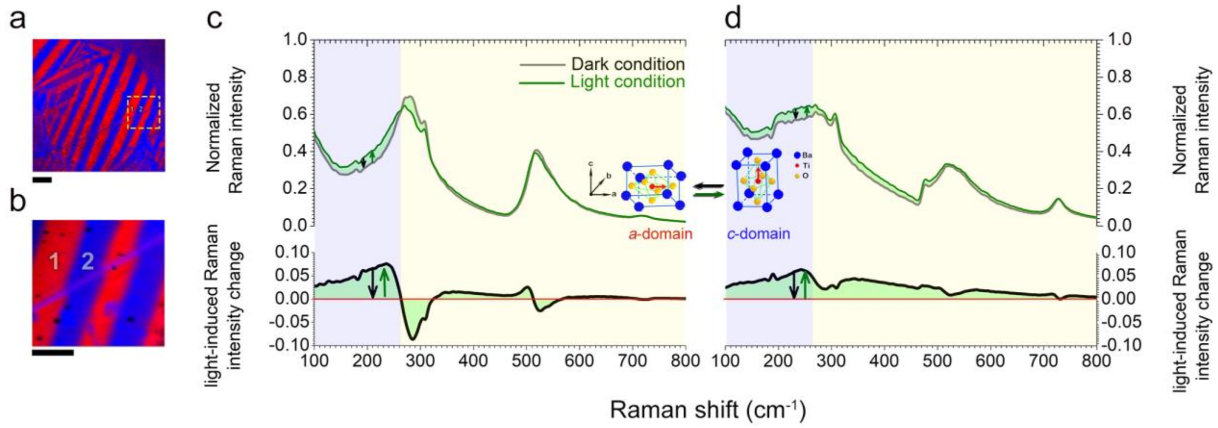

Figure S7 | Influence of the lattice-charge coupling in the photo- response phenomena of polycrystalline ferroelectrics. a-b, Raman image (panel **a**) and its magnification (panel **b**) of the surface coarse-grained BTO sample. Scale bars, 10 and 1 µm, respectively. The regions denoted as **1** and **2** in the panel **b** show the positions where the characteristic Raman spectra corresponding to *a*-domain, i.e. in-plane polarization (red region in the panels a-b), and *c*-domain, i.e. out-of-plane polarization (blue region of the panels a-b) of BTO are taken for each domain under dark and illumination conditions (that is, under a light power of 40 mW). **c**-**d**, Average Raman spectra of *a*-domain (panel **c**) and *c*-domain (panel **d**) of coarse-grained BTO under dark and illumination conditions. In both cases, the average Raman spectra are normalized by taking into account the value in the dark condition. Additionally, the calculated light induced Raman intensity change for each domain is plotted at bottom of each image. The difference of light-induced Raman intensity change is represented by the green region in the panel **c** and **d**. The purple and yellow areas delimit the Raman mode regions where is located the Ba-O bond (A-site) and Ti-O bonds(B-site) of the perovskite structure, respectively.

Supplementary References

1. Pezzoti, P.; Okai, K.; Zhu, W. Stress Tensor Dependence of the Polarized Raman Spectrum of Tetragonal Barium Titanate. *J. Appl. Phys.* **2012**, *111*, 013504.
2. Rubio-Marcos, F.; Del Campo, A.; Marchet, P.; Fernández, J. F. Ferroelectric Domain Wall Motion Induced by Polarized Light. *Nat. Commun*. **2015**, *6*, 6594.
3. DiDomenico, M.; Wemple, S.H.; Porto, S.P.S.: Bauman, R.P. Raman Spectrum of Single-Domain $BaTiO_3$. *Phys. Rev.* **1968**, *174*, 522-530.
4. Venkateswaran, U.D.; Naik, V.M.; Naik, R. High-Pressure Raman Studies of Polycrystalline $BaTiO_3$. *Phys. Rev. B* **1998**, *58*, 14256.
5. Dobal, P.S.; Katiyar. R.S. Studies on Ferroelectric Perovskites and Bi-Layered Compounds using Micro-Raman Spectroscopy. *J. Raman Spectrosc.* **2002**, *33*, 405-423.
6. Shiratori, Y.; Pithan, C.; Dornseiffer. J.; Waser. R. Raman Scattering Studies on Nanocrystalline $BaTiO_3$ Part I – Isolated Particles and Aggregates. *J. Raman Spectrosc.* **2007**, *38*, 1288-1299.
7. Shiratori, Y.; Pithan, C.; Dornseiffer. J.; Waser. R. Raman Scattering Studies on Nanocrystalline $BaTiO_3$ Part II – Consolidated Polycrystalline Ceramics *J. Raman Spectrosc.* **2007**, *38*, 1300-1306.
8. Pinczuk, A.; Taylor, W.T.; Burstein, E.; Lefkowitz, I. The Raman Spectrum of $BaTiO_3$*. Solid State Commun.* **1967**, *5*, 429-433.
9. Burns, G.; Scott, B.A. Raman Scattering in the Ferroelectrics System $Pb_{1-x}Ba_xTiO_3$. *Solid State Commun.* **1971**, *9*, 813-817.
10. Sun, Y.; Abid, A.Y.; Tan, C.; Ren, C.; Li, M.; Li, N.; Chen, P.; Li, Y.; Zhang, J.; Zhong, X.; Wang, J.; Liao, M.; Liu, K.; Bai, X.; Zhou, Y.; Yu, D.; Gao, P. Subunit Cell–Level Measurement of Polarization in an Individual Polar Vortex. *Sci. Adv.* **2019**, **5**, eaav4355.
11. Dietzek, B.; Cialla, D.; Schmitt, M.; Popp, J. Introduction to the Fundamentals of Raman Spectroscopy. Confocal Raman Microscopy. Second Edition. (ed. Toporski, J.; Thomas Dieing, T.; Hollricher, O.) Chapter ***3***, 47-68 (Springer Nature, 2018)
